# Therapeutic Plasma Exchange in the Elderly: Rare Indications but Good Tolerability

**DOI:** 10.1002/jca.70155

**Published:** 2026-06-22

**Authors:** Valentin Coirier, Quentin Quelven, Pauline Guillot, Elie Jalaber, Floriane L’Her, Vincent Joussellin, Félicie Bélicard, Kieran Pinceaux, Adel Maamar, Mathieu Lesouhaitier, Jean‐Marc Tadié, Nicolas Terzi

**Affiliations:** ^1^ Médecine Intensive‐Réanimation CHU de Rennes Rennes France; ^2^ Faculté de Médecine Université de Rennes 1 Rennes France

**Keywords:** adverse event, elderly, therapeutic plasma exchange

## Abstract

Therapeutic plasma exchange (TPE) is used for a wide range of indications and is associated with well‐described adverse events. However, no study has specifically focused on tolerance in elderly patients, despite its expected increasing use in this population. We conducted a single‐center retrospective case–control study of all patients aged ≥ 75 years who underwent TPE between 2011 and 2023. Adverse events were identified through standardized surveillance forms completed contemporaneously. The primary outcome was the rate of any adverse event occurring during TPE sessions, compared between patients aged ≥ 75 years (elderly group) and those < 75 years. Elderly patients accounted for 7.4% of all TPE sessions during the study period. Thirty‐one patients were included in each group, corresponding to 506 TPE sessions. Hypertension and cardiac disease were significantly more frequent in the elderly group. Overall adverse event rates were similar in the two groups (40.6% in < 75 years vs. 43.4% in ≥ 75 years, *p* = 0.592). Asymptomatic hypocalcemia was more frequent in elderly patients, whereas allergic reactions were more frequent in younger patients. One‐year survival did not differ between groups (*p* = 0.31). TPE in patients aged ≥ 75 years is uncommon, but its tolerance is comparable to that of younger patients. When clinically indicated, TPE should not be withheld on the basis of age alone.

**Abbreviations**
ACD‐A: acid citrate dextrose solution A
FFP: fresh frozen plasma
TPE: therapeutic plasma exchange
UERTU: Urgent Extracorporeal Replacement Therapy Unit

## 1 Introduction

Therapeutic plasma exchange (TPE) was first introduced into clinical practice in the late 1950s [1]. Since then, its efficacy has been extensively evaluated [2, 3, 4], and international guidelines with levels of evidence are regularly updated [5]. The exact mechanism of action of TPE is not fully understood [6]. It is generally accepted, however, that its therapeutic effects rely mainly on two processes: the removal of pathogenic substances from the circulation [3, 7] and/or the replacement of deficient plasma components [8]. TPE is currently performed either with high‐permeability filters integrated into standard multifunctional renal replacement systems or with dedicated centrifugation devices [9].

The incidence of adverse events during TPE sessions has been reported in recent years, with variability across studies ranging from 7.6% to 42.5% of sessions [10, 11, 12]. Among these, hypotension is one of the most frequent adverse events, occurring in up to 15.2% of sessions in some series [11]. Advances in modern medicine have markedly improved survival and transformed a number of previously fatal diseases into chronic, manageable conditions. In this context, the indications for TPE in elderly patients are expected to increase over time, as several of the conditions requiring TPE are diseases whose incidence rises with age [13, 14]. However, advanced age is associated with a greater burden of comorbidities—per one cross‐sectional study, the mean number of comorbidities in patients between 65 and 84 years of age was 2.6, and the mean number of comorbidities in patients 85 years of age or older was 3.62 [15]. Moreover, the frailty of older patients makes them more vulnerable to acute medical events. Therefore, population‐aging [16] will likely result in a growing number of elderly individuals requiring TPE, while their frailty may predispose them to reduced tolerance of this treatment. In the absence of studies specifically addressing the safety and tolerance of TPE in this population, we considered it necessary to conduct such an investigation.

## 2 Materials and Methods

### 2.1 Study Design and Patients

We conducted a single‐center retrospective case–control study at Rennes University Hospital between January 2011 and December 2023. Eligibility criteria for the elderly group were: adults aged ≥ 75 years [17] who had undergone at least one TPE session during the study period. For each patient included in the elderly group, one control patient was selected in a 1:1 ratio. Eligibility criteria for the control group were: adults aged < 75 years who had undergone at least one TPE session during the same period. Matching was performed according to the indication for TPE. Patients under legal protection, minors, those who declined to participate, and those for whom monitoring forms were unavailable were excluded. The study protocol was approved by the Rennes University Hospital Institutional Review Board (agreement number 23.94) prior to study initiation.

### 2.2 TPE Sessions

All TPE sessions were performed by nurses specifically trained in the technique within the Urgent Extracorporeal Replacement Therapy Unit (UERTU) of the Medical Intensive Care Department. Procedures were usually carried out in the dedicated TPE room of the Medical Intensive Care Department. Patients hospitalized in other wards were generally transferred to this room, except for critically ill patients in other Intensive Care Units, for whom TPE was performed at the bedside.

The indication and number of sessions were determined according to international recommendation [5, 18, 19, 20, 21], in agreement with the patient's referring physician and the TPE‐referent intensivist. The choice of replacement fluid (Fresh Frozen Plasma [FFP] or 4% albumin) depended on the underlying indication, bleeding risk, and interval between sessions. The type and number of FFP units were supplied by the French Blood Establishment (Établissement Français du Sang), subject to stock availability.

Centrifugation was the sole technique used in our center, regardless of indication, because of its higher plasma extraction efficiency compared with filtration and its feasibility with peripheral venous access [9]. The device employed was the Comtec Cell Separator (Fresenius Kabi, 61 352 Bad Homburg, Germany). Anticoagulation of the extracorporeal circuit was achieved with acid citrate dextrose solution A (ACD‐A). The total volume of ACD‐A and the volume reinfused were automatically calculated by the device. To prevent hypocalcemia, calcium supplementation was administered continuously during the procedure, with the dosage adapted according to baseline ionized calcium levels and the type of replacement fluid. In general, 2 g/h of calcium gluconate was infused when albumin was used, and 2 g/h of calcium chloride when FFP was administered.

The choice of vascular access depended on the indication for TPE, the number of sessions planned, the patient's clinical status, and the condition of the peripheral veins. When required, ultrasound‐guided insertion of a dialysis catheter into the jugular or subclavian vein was performed, with systematic chest radiography to confirm correct positioning and exclude complications such as pneumothorax.

Standardized monitoring forms were designed for each TPE session and were prospectively completed by the nursing staff.

### 2.3 Data Collection

Patient characteristics were collected, including age, height, weight, comorbidities, and TPE indication. Cardiorespiratory monitoring parameters during sessions were recorded every 15 min (blood pressure, heart rate, pulse oximetry, and respiratory rate), as well as data related to the TPE procedures (volumes processed and treated, nature and site of venous access, hypocalcemia or anaphylaxis symptoms, ACD‐A volume used, dose of calcium infused, device‐related problems, discontinuation of the session, and any other event considered to be an adverse effect). Biological data collected included serum ionized calcium levels, pH, and hematocrit. Catheters were sent for culture only in cases of suspected infection. Bacteriological results of the catheter culture were collected, as well as all blood cultures performed during the treatment period.

### 2.4 Adverse Effects: Definitions and Classifications

Hypotension was defined as a mean arterial pressure < 65 mmHg and/or a decrease in systolic arterial pressure > 30 mmHg from baseline during the session, and was classified into three grades of severity (Supporting Information 1). Hypocalcemia was diagnosed when the serum ionized calcium level was < 1.10 mmol/L, with severity determined according to the Lee classification [22]. Allergic reactions were graded from I to IV according to the Ring and Messmer classification [23].

Complications related to venous access (e.g., pain, bleeding at the insertion site, catheter dysfunction, issues with single‐use kits, or centrifuge malfunction) were recorded from standardized monitoring forms. Chest X‐rays performed after dialysis catheter insertion at the superior vena cava site were systematically reviewed to confirm proper positioning and exclude pneumothorax. Venous Doppler ultrasound was performed if catheter‐related thrombosis was suspected. The catheter tip was sent for culture in cases of local inflammation or systemic signs of infection. The diagnosis of catheter‐related infection was established in accordance with international recommendations [24].

### 2.5 Endpoints and Statistical Analysis

The primary endpoint was the rate of all‐type adverse effects, expressed as the number (percent) of sessions and the number (percent) of patients. The rates of each type of adverse effect and one‐year survival rate were secondary endpoints. Proportions were estimated with the 95% confidence interval (95% CI) by the method of Clopper and Pearson. Statistical analysis was performed using Rstudio version 4.2.3. Quantitative variables were expressed as medians (25th percentile–75th percentile) and comparisons between groups were performed using the Mann Whitney *U* test or Kruskall–Wallis test. Categorical variables were compared using the chi‐square test or Fisher's exact test, when appropriate. Overall survival (from the first TPE session) comparison was realized using a log‐rank test. A generalized estimating equations (GEE) model was used to account for within‐patient correlation due to repeated sessions. All tests were two‐sided and a *p* value < 0.05 was considered statistically significant.

## 3 Results

### 3.1 Description of the Population and TPE Sessions

**FIGURE 1:**
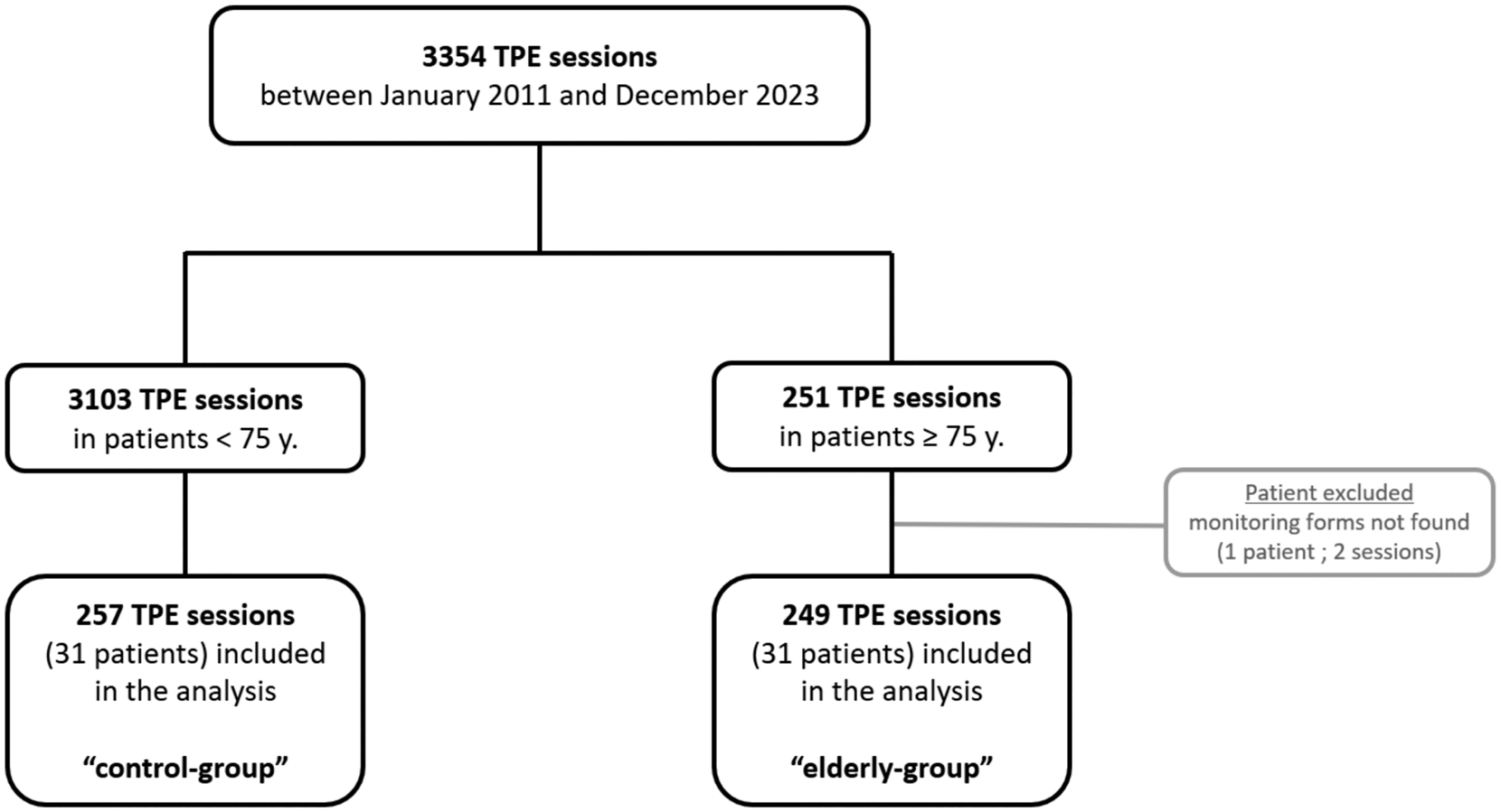
Flow chart. Abbreviation: TPE: therapeutic plasma exchange.

Between January 2011 and December 2023, 251 TPE sessions were performed in patients aged ≥ 75 years, representing 7.4% (251/3354) of all procedures during this period (Figure 1). Of these, 249 sessions were included in the analysis, corresponding to 31 patients in the elderly group. These patients were matched with 31 younger individuals in the control group, who underwent 257 TPE sessions. The matching between the two patient groups was not strictly identical regarding hyperviscosity syndrome, Guillain–Barré syndrome, focal segmental glomerulosclerosis, and central neurological disorders (Supporting Information 2). The two groups showed similar median numbers of TPE sessions (Table 1), but comorbidities were significantly more frequent in the elderly group; notably hypertension (58.1% [40.7%–75.5%] vs. 9.7% [2.0%–25.8%], *p* < 0.001) and cardiac disease (29% [13.0%–45.0%] vs. 3.2% [0.1%–16.7%], *p* = 0.012). The use of a dialysis catheter was also more common in elderly patients (74.2% [58.8%–89.6%] vs. 45.2% [27.7%–62.7%], *p* = 0.038).

**TABLE 1:** Patients characteristics.

	All patients (*N* = 62)	< 75 years (*N* = 31)	≥ 75 years (*N* = 31)	*p*
Age (years)	75 [50–78]	50 [32–58]	78 [77–81]	< 0.001
Body Mass Index (kg m^−2^)	24.7 [21.6–26.7]	23.3 [20.1–26.6]	25.2 [22.8–27.9]	0.083
Number of TPE sessions	5 [3–7]	5 [3–7]	4 [3–7]	0.676
Sex (female)	33 (53.2%)	18 (58.1%)	15 (48.4%)	0.611
Comorbidities				
Hypertension	21 (33.9%)	3 (9.7%)	18 (58.1%)	< 0.001
Cardiac disease	10 (16.1%)	1 (3.2%)	9 (29%)	0.012
Respiratory disease	5 (8.1%)	3 (9.7%)	2 (6.5%)	> 0.9
Chronic kidney disease	2 (3.2%)	1 (3.2%)	1 (3.2%)	1.0
Neoplastic disease	21 (33.9%)	8 (25.8%)	13 (41.9%)	0.283
Venous access				0.07
Dialysis catheter	37 (59.7%)	14 (45.2%)	23 (74.2%)	0.038
Peripheral venous catheter	21 (33.9%)	14 (45.2%)	7 (22.6%)	0.107
Canaud catheter	4 (6.5%)	3 (9.7%)	1 (3.2%)	0.612

Abbreviation: TPE: therapeutic plasma exchange.

The three main indications (Table 2) were thrombotic microangiopathy other than thrombotic thrombocytopenic purpura (19.4% of the patients, *N* = 12), peripheral neuropathy (16.1% of patients, *N* = 10), and hyperviscosity syndrome (14.5% of patients, *N* = 9). Session duration was longer in younger patients (107 min [97–123]) compared with elderly patients (98 min [89–109], *p* < 0.001) (Table 3). Prescribed volumes, adjusted for body weight, were similar between groups (53.6 mL/kg [48.9–63.6] in controls vs. 50 mL/kg [49.3–57.1] in elderly patients, *p* = 0.113). FFP was more frequently used in the control group (34% [28.2%–39.8%] vs. 22.5% [17.3%–27.7%] of sessions, *p* = 0.006), whereas 4% albumin was more often used in the elderly group (73.9% [68.4%–79.4%] vs. 60.2% [54.2%–66.2%], *p* = 0.001). Younger patients also received larger amounts of ACD‐A (97 mL/session [65–432] vs. 77 mL [62–106], *p* < 0.001) and calcium supplementation (11.3 mmol/session [9.1–13.6] vs. 7.7 mmol/session [6.1–9.1], *p* < 0.001). At baseline, mean arterial pressure and heart rate were comparable between groups.

**TABLE 2:** Therapeutic plasma exchange indications.

Indications	TPE sessions			
	All sessions (*N* = 506)	< 75 years (*N* = 257)	≥ 75 years (*N* = 249)	*p*
TMA, non‐TTP	76 (15.0%)	48 (18.8%)	28 (11.2%)	0.026
Peripheral neuropathy	169 (33.5%)	35 (13.7%)	134 (53.8%)	< 0.001
Hyperviscosity syndrome	18 (3.6%)	8 (3.1%)	10 (4.0%)	0.764
TTP	51 (10.1%)	29 (11.3%)	22 (8.8%)	0.434
Myasthenia gravis	27 (5.3%)	12 (4.7%)	15 (6.0%)	0.639
Cryoglobulinemia	19 (3.8%)	10 (3.9%)	9 (3.6%)	> 0.9
ANCA‐associated vasculitis	26 (5.1%)	14 (5.5%)	12 (4.8%)	0.898
Guillain‐Barre syndrome	12 (2.4%)	0 (0%)	12 (4.8%)	< 0.001
Transverse myelitis	7 (1.4%)	4 (1.6%)	3 (1.2%)	> 0.9
Encephalitis	4 (0.8%)	4 (1.6%)	0 (0%)	0.124
Hemolytic anemia	4 (0.8%)	3 (1.2%)	1 (0.4%)	0.624
Acute liver failure	6 (1.2%)	3 (1.2%)	3 (1.2%)	1.000
Multiple sclerosis	5 (1.0%)	5 (2.0%)	0 (0%)	0.061
FSGS	77 (15.2%)	77 (30.1%)	0 (0%)	< 0.001

Abbreviations: ANCA: anti‐neutrophil cytoplasmic antibodies; FSGS: focal segmental glomerulosclerosis; TMA: thrombotic microangiopathy; TPE: therapeutic plasma exchange; TTP: thrombotic thrombocytopenic purpura.

**TABLE 3:** Therapeutic plasma exchange sessions characteristics.

	All sessions (*N* = 506)	< 75 years (*N* = 257)	≥ 75years (*N* = 249)	*p*
Duration (minutes)	102.0 [92.0–118.0]	107.0 [97.0–123.2]	98.0 [89.0–109.0]	< 0.001
Volumes, replacements fluids, anticoagulation and calcium				
Prescribed volume (mL kg^−1^)	50.0 [48.9–60.3]	53.6 [48.9–63.6]	50.0 [49.3–57.1]	0.113
Prescribed volume (mL)	3800 [3000–4500]	4450 [3500–4500]	3600 [3000–4000]	< 0.001
Volume really substituted (mL)	3787 [3164–4381]	4291 [3590–4461]	3563 [2934–3862]	< 0.001
Ratio exchanged volume/plasma volume	1.3 [1.2–1.4]	1.3 [1.1–1.4]	1.3 [1.2–1.4]	0.069
FFP as fluid replacement alone	143 (28.3% [24.4%–32.2%])	87 (34.0% [28.2%–39.8%])	56 (22.5% [17.3%–27.7%])	0.006
Albumin 4% as fluid replacement alone	338 (66.9% [62.8%–71.1%])	154 (60.2% [54.2%–66.2%])	184 (73.9% [68.4%–79.4%])	0.001
Mixed fluid replacement (albumin + FFP)	24 (4.8% [2.9%–6.7%])	15 (5.9% [3.0%–8.8%])	9 (3.6% [1.3%–5.9%])	0.329
Volume of ACD‐A used in procedure (mL)	534 [479–611]	569 [490–641]	523 [476–567]	< 0.001
Volume of ACD‐A reinfused (mL)	81 [63–386]	97 [65–432]	77 [62–106]	< 0.001
Calcium infused during session (mmol)	9.1 [7.0–11.3]	11.3 [9.1–13.6]	7.7 [6.1–9.1]	< 0.001
Hemodynamic characteristics				
Baseline systolic arterial pressure (mmHg)	136 [121–146]	134 [116–143]	137 [126–150]	< 0.001
Baseline diastolic arterial pressure (mmHg)	69 [60–79]	73 [61–81]	67 [60–75]	< 0.001
Baseline mean arterial pressure (mmHg)	92 [83–100]	93 [79–101]	91 [84–97]	0.272
Baseline heart rate (/min)	77 [67–88]	76 [67–90]	78 [66–87]	0.899
Decrease in systolic arterial pressure from baseline (mmHg)	8.5 [0.0–18.0]	7.0 [0.0–14.0]	10.0 [0.0–22.0]	0.005
Lowest mean arterial pressure (mmHg)	81 [71–89]	84 [71–92]	79 [71–85]	< 0.001
Lowest systolic arterial pressure (mmHg)	124 [110–134]	124 [107–133]	123 [111–135]	0.410
Lowest diastolic arterial pressure (mmHg)	61 [52–68]	65 [55–72]	58 [51–63]	< 0.001
Biological characteristics				
pH before session	7.4 [7.4–7.4]	7.4 [7.4–7.4]	7.4 [7.4–7.4]	0.477
pH after session	7.3 [7.3–7.4]	7.3 [7.3–7.4]	7.3 [7.3–7.4]	0.432
Ionized calcium before session (mmol/L)	1.2 [1.1–1.2]	1.2 [1.1–1.2]	1.2 [1.1–1.2]	0.239
Ionized calcium after session (mmol/L)	1.1 [1.1–1.2]	1.2 [1.1–1.3]	1.1 [1.1–1.2]	< 0.001
Hematocrit before session (%)	31 [27–37.0]	30 [27–33]	33 [28–39]	< 0.001
Hematocrit after session (%)	31 [27–36]	30 [27–33]	33 [28–38]	< 0.001

Abbreviations: ACD‐A: acid citrate dextrose solution A; FFP: fresh frozen plasma.

### 3.2 Description of Adverse Events

Overall adverse event rates did not differ significantly between the groups, whether analyzed per session (40.6% [37.7%–77.2%] in younger vs. 43.4% [37.2%–49.6%] in elderly, *p* = 0.592) (Table 4) or per patient (90.3% [79.9%–100%] vs. 87.1% [75.3%–98.9%], *p* > 0.9) (Supporting Information 3). The most frequent complication was hypotension, occurring in 21.7% [16.6%–26.8%] of elderly sessions versus 16.4% [11.9%–20.9%] of younger sessions (*p* = 0.162). During sessions, the decrease in systolic blood pressure was greater in elderly patients (10 mmHg [0.0–22.0] vs. 7 mmHg [0.0–14.0], *p* = 0.005), and their lowest mean arterial pressure was also lower (79 mmHg [71–85] vs. 84 mmHg [71–92], *p* < 0.001). In both groups, hypotension was mainly asymptomatic (93% of cases), symptomatic in 1.2% [0.3%–2.1%] of sessions, and severe in only one case (elderly group).

**TABLE 4:** Complications during TPE sessions.

	All sessions (*N* = 506)	< 75 years (*N* = 257)	≥ 75 years (*N* = 249)	*p*
All complications	212 (42.0% [37.7%–46.3%])	104 (40.6% [36.8%–77.2%])	108 (43.4% [37.2%–49.6%])	0.592
Hypotension	96 (19.0% [15.6%–22.4%])	42 (16.4% [11.9%–20.9%])	54 (21.7% [16.6%–26.8%])	0.162
Asymptomatic hypotension	89 (17.6% [14.3%–20.9%])	40 (15.6% [11.1%–20.0%])	49 (19.7% [14.8%–24.6%])	0.281
Non severe symptomatic hypotension	6 (1.2% [0.3%–2.1%])	2 (0.8%)	4 (1.6% [0.04%–3.2%])	0.444
Severe symptomatic hypotension	1 (0.2%)	0 (0.0%)	1 (0.4%)	0.493
Hypocalcemia	81 (16.0% [12.8%–19.2%])	36 (14.1% [9.8%–18.4%])	45 (18.1% [13.3%–22.9%])	0.269
Asymptomatic hypocalcemia	70 (13.9% [10.9%–16.9%])	27 (10.5% [6.8%–14.2%])	43 (17.3% [12.6%–22.0%])	0.040
Hypocalcemia Grade I	9 (1.8% [0.6%–3.0%])	7 (2.7% [0.7%–4.7%])	2 (0.8%)	0.176
Hypocalcemia Grade II	2 (0.4%)	2 (0.8%)	0 (0%)	0.499
Hypocalcemia Grade III	0 (0%)	0 (0%)	0 (0%)	1
Allergic reaction	16 (3.2% [1.7%–4.7%])	13 (5.1% [2.4%–7.8%])	3 (1.2%)	0,020
Grade I	15 (3.0% [1.5%–4.5%])	12 (4.7% [2.1%–7.3%])	3 (1.2%)	0,033
Grade II	1 (0.2%)	1 (0.4%)	0 (0%)	1
Grade III	0 (0%)	0 (0%)	0 (0%)	1
Venous access complication	48 (9.5% [6.9%–12.1%])	22 (8.6% [5.2%–12.3%])	26 (10.4% [6.6%–14.2%])	0.578
Desaturation	5 (1.0% [0.1%–1.9%])	5 (2.0%)	0 (0%)	0.061
Digestive symptoms	3 (0.6%)	3 (1.2%)	0 (0%)	0.249
Other complication	29 (5.7% [3.7%–7.7%])	19 (7.4% [4.2%–10.6%])	10 (4.0% [1.6%–6.4%])	0.146

Hypocalcemia occurred in 16% [12.8%–19.2%] of sessions and in 61.3% [49.2%–73.4%] of patients. Asymptomatic hypocalcemia was more frequent in the elderly population (17.3% [12.6%–22.0%] vs. 10.5% [6.8%–14.2%] of sessions, *p* = 0.04). Symptomatic hypocalcemia occurred in 1.8% [0.6%–3.0%] of sessions (Grade I) and 0.4% of sessions (Grade II).

Allergic reactions occurred in 3.2% [1.7%–4.7%] of sessions and in 17.7% [8.2%–27.2%] of patients, and were more frequent in younger patients (5.1% vs. 1.2% of sessions, *p* = 0.02). Most reactions were Grade I (94% [82.4%–100%]), with a single Grade II reaction reported.

Other complications, mainly related to venous access, did not differ between the groups (Table 4). No association was found between the number of sessions and complication rates in the GEE model (Supporting Information 4).

### 3.3 Overall Survival

**FIGURE 2:**
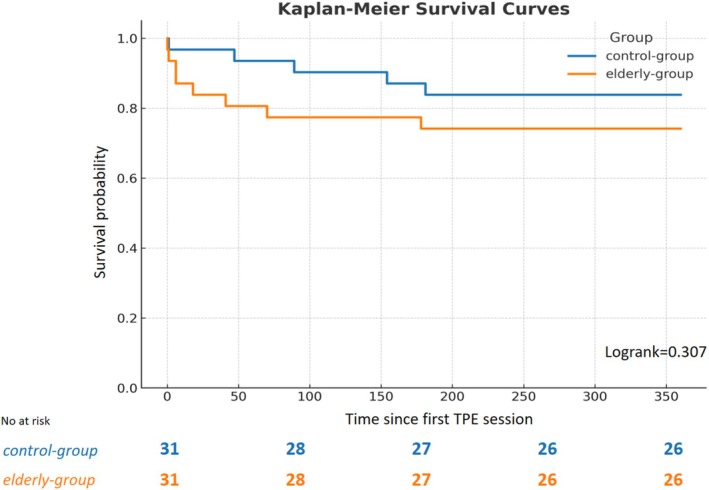
Survival curves of the overall population according to the age (< 75 vs. ≥ 75). Abbreviation: TPE: therapeutic plasma exchange.

One‐year survival was not significantly different between the two groups: 83.9% in younger patients and 74.2% in elderly patients (*p* = 0.307) (Figure 2).

## 4 Discussion

To our knowledge, this is the first study to specifically assess the tolerance of TPE in patients aged ≥ 75 years. The main finding is that TPE was well tolerated in this population, with no significant difference in the occurrence of adverse events compared with younger patients. It should nevertheless be noted that allergic reactions were more frequent among younger patients. Although indications for TPE remain not frequent in the elderly, the aging of the population suggests that such indications will become increasingly common in the coming years. Our findings indicate that advanced age alone should not be considered a barrier to TPE when clinically indicated.

Previous studies have evaluated tolerance according to age; however, all compared patients aged ≥ 65 years with those < 65 years [25, 26, 27, 28, 29, 30]. While physiological age is a key determinant, the threshold commonly used in Western countries to define an elderly population is 75 years rather than 65 years [17]. Despite these differences in age‐group definitions, available data—including ours—have not shown any major difference in tolerance between older and younger patients [29, 30].

An unexpected finding of our study concerns the good hemodynamic tolerance. We observed a greater decrease in systolic blood pressure and a lower minimal mean arterial pressure during sessions in the elderly group. However, this difference, although statistically significant, was not clinically meaningful (with no impact on therapeutic management) and did not meet the predefined criteria for hypotension. A higher incidence of hypotension could have been anticipated, given the age‐related cardiovascular modifications and its consequences [31], but this was not observed. To note, antihypertensive therapy was not specifically discontinued during plasma exchange sessions.

Although overall rates of hypocalcemia did not differ significantly between groups, asymptomatic hypocalcemia was more frequent in elderly patients. Baseline calcium levels were comparable, but post‐treatment levels were lower in the elderly group. A direct age‐related mechanism is not evident, even though hypocalcemia is more common in elderly hospitalized patients [32]. In our practice, calcium supplementation is related to the type of replacement fluid used during TPE procedures. However, this does not explain the observed difference between groups. While differences in replacement fluid exposure may have contributed, this finding likely reflects additional factors and cannot be fully explained based on the available data. While hypocalcemia remained largely asymptomatic, these results suggest that closer monitoring of calcium levels in elderly patients may be advisable.

Finally, the one‐year survival did not differ significantly between the two groups, in contrast to a previous study reporting higher mortality in patients aged ≥ 65 years undergoing TPE [29]. In that study, one‐year mortality was higher in patients older than 65 years despite similar one‐month mortality, suggesting that deaths were more likely related to underlying disease rather than to the TPE procedure itself. This difference was observed only in neurologic indications, possibly because they were the most frequent indication and/or because neurologic diseases may result in long‐term sequelae associated with additional complications. These findings were not observed in our study, possibly due to limited statistical power or improvements in the management of these conditions over time. Although our sample size was limited, this finding is noteworthy as it highlights the non‐futility of performing TPE in elderly patients.

This study has several limitations that should be acknowledged. First, the relatively small sample size (*n* = 31 per group) limits the statistical power of the analysis, particularly for survival outcomes and subgroup analyses, and therefore negative findings should be interpreted with caution. Second, although matching was performed, it did not fully balance all baseline characteristics and clinical indications, which may have resulted in residual confounding. In addition, no multivariable adjustment could be conducted due to the limited sample size, further restricting the ability to control for potential baseline imbalances. Third, the retrospective design of the study introduces an inherent risk of selection bias, as only patients considered suitable for TPE were included, thereby limiting the generalizability of the findings. Nevertheless, our results provide a reassuring message regarding both the tolerance and the potential benefit of this therapeutic approach in older patients.

In conclusion, indications for TPE in patients aged ≥ 75 years remain uncommon, but tolerance did not differ significantly between groups. When clinically justified, TPE should not be withheld from this age group. However, these findings should be interpreted with caution given the limited sample size.

## Funding

The authors have nothing to report.

## Conflicts of Interest

The authors declare no conflicts of interest.

## Supporting information


**Supporting Information 1:** Adverse event classification.

**Supporting Information 2:** TPE indications in two groups.

**Supporting Information 3:** Therapeutic plasma exchange‐related complications per patient according to study group.

**Supporting Information 4:** Description and results of the generalized estimating equations model.

## Data Availability

The data that support the findings of this study are available from the corresponding author upon reasonable request.
