# Supplementary material for: Therapeutic Plasma Exchange in the Elderly: Rare Indications but Good Tolerability
Source: J Clin Apher. 2026 Jun 22;41(3):e70155. doi: 10.1002/jca.70155 (PMC13287298; doi:10.1002/jca.70155)
Supplement: Supplementary file 1 — Supporting Information 1: Adverse event classification. [file JCA-41-e70155-s002.docx]

**Supplementary material 1. Adverse event classification.**

**Hypotension**

Once the diagnosis of hypotension was made, it was classified according to severity:

- grade I: asymptomatic hypotension if there were no associated symptoms and no specific therapy was given
- grade II: non-severe symptomatic hypotension if there was a need for volume expansion or increasing the dose of a vasoactive agent
- grade III: severe symptomatic hypotension if TPE session had to be stopped because of low blood pressure

**Hypocalcemia**

The severity of hypocalcemia was determined according to the Lee classification:

- grade I: asymptomatic (symptomless hypocalcemia)
- grade II: mild (perioral and/or distal paresthesia, sneezing, dizziness, flushes, chills, headache)
- grade III: moderate (nausea/vomiting, irritability, abdominal cramps, spasms, hypotension, tremor)
- grade IV: severe (heart rhythm disorders, attack)

**Allergic reactions**

The severity of allergic reactions was determined according to the Ring and Messmer classification:

- grade I: general skin signs (erythema, urticaria, edema)
- grade II: moderate multi-visceral disorders (skin/mucosal signs, hypotension or unusual tachycardia, bronchial hyperreactivity)
- grade III: life threatening multi-visceral disorders that required specific therapy (collapse, tachycardia or bradycardia, heart rhythm disorders, bronchospasm)
- grade IV: allergic reactions complicated by cardiac arrest
