# Supplementary material for: Therapeutic Plasma Exchange in the Elderly: Rare Indications but Good Tolerability
Source: J Clin Apher. 2026 Jun 22;41(3):e70155. doi: 10.1002/jca.70155 (PMC13287298; doi:10.1002/jca.70155)
Supplement: Supplementary file 2 — Supporting Information 2: TPE indications in two groups. [file JCA-41-e70155-s003.docx]

**Supplementary material 2. TPE indications in two groups.**

| **Indications** | **<75 y (n=31)** | **≥75 y (n=31)** |
| --- | --- | --- |
| Thrombotic microangiopathy, non-TTP | 6 | 6 |
| Hyperviscosity syndrome | 4 | 5 |
| Peripheral neuropathy | 5 | 5 |
| Thrombotic thrombocytopenic purpura | 3 | 3 |
| Myasthenia gravis | 3 | 3 |
| Cryoglobulinemia | 2 | 2 |
| ANCA-associated vasculitis | 2 | 2 |
| Guillain barré syndrome | 0 | 2 |
| Hemolytic anemia | 1 | 1 |
| Transverse myelitis | 1 | 1 |
| Acute Liver Failure | 1 | 1 |
| Focal Segmental Glomeruloscloris | 1 | 0 |
| Multiple sclerosis | 1 | 0 |
| Encephalitis | 1 | 0 |
