# Supplementary material for: Therapeutic Plasma Exchange in the Elderly: Rare Indications but Good Tolerability
Source: J Clin Apher. 2026 Jun 22;41(3):e70155. doi: 10.1002/jca.70155 (PMC13287298; doi:10.1002/jca.70155)
Supplement: Supplementary file 3 — Supporting Information 3: Therapeutic plasma exchange‐related complications per patient according to study group. [file JCA-41-e70155-s001.docx]

Complications during TPE sessions

|  | **Patients** | | | |
| --- | --- | --- | --- | --- |
|  | **All patients (N=66)** | **<75 y (N=33)** | **≥75 y (N=33)** | **p-value** |
| All complications | 55 (88.7%) | 28 (90.3%) | 27 (87.1%) | >0.9 |
| **Hypotension** | 29 (46.8%) | 14 (45.2%) | 15 (48.4%) | 0.8 |
| - Asymptomatic hypotension | 27 (43.5%) | 14 (45.2%) | 13 (41.9%) |  |
| - Non severe symptomatic hypotension | 3 (4.8%) | 1 (3.2%) | 2 (6.5%) |  |
| - Severe symptomatic hypotension | 1 (1.6%) | 0 | 1 (3.2%) |  |
| **Hypocalcemia** | 38 (61.3%) | 19 (61.3%) | 19 (61.3%) | >0.9 |
| - Asymptomatic hypocalcemia | 34 (54.8%) | 15 (48.4%) | 19 (61.3%) |  |
| - Hypocalcemia grade I | 9 (14.5%) | 7 (22.6%) | 2 (6.5%) |  |
| - Hypocalcemia grade II | 2 (3.2%) | 2 (6.5%) | 0 |  |
| - Hypocalcemia grade III | 0 | 0 | 0 |  |
| **Allergic reaction** | 11 (17.7%) | 8 (25.8%) | 3 (9.7%) | 0.11 |
| - Grade I | 11 (17.7%) | 8 (25.8%) | 3 (9.7%) |  |
| - Grade II | 1 (1.6%) | 1 (3.2%) | 0 |  |
| - Grade III | 0 | 0 | 0 |  |
| Venous Access complication | 22 (35.5%) | 13 (41.9%) | 9 (29%) | 0.3 |
| Desaturation | 2 (3.2%) | 2 (6.5%) | 0 | 0.5 |
| Digestive symptoms | 4 (6.5%) | 4 (12.9%) | 0 | 0.11 |
| Other complication | 12 (19.4%) | 6 (19.4%) | 6 (19.4%) | >0.9 |
