# Supplementary material for: Therapeutic Plasma Exchange in the Elderly: Rare Indications but Good Tolerability
Source: J Clin Apher. 2026 Jun 22;41(3):e70155. doi: 10.1002/jca.70155 (PMC13287298; doi:10.1002/jca.70155)

GEE model

To ensure the robustness of our results, we performed an additional analysis using a generalized estimating equations (GEE) model, allowing us to account for within-patient correlation and to examine the association between age, session number, and the occurrence of complications. We tested also the interaction between age and the session number on the occurrence of complications.

After adjusting for the session number, there was no significant association between old age (age ≥75 years) and complications (OR ≈ 1.005, 95% CI [0.574-1.736], p = 0.987). The session number showed a trend towards a lower risk of complications with increasing sessions (OR ≈ 0.891 per session, 95% CI [0.793–1.001], p = 0.051), but this did not reach statistical significance. The interaction between old age and session number was not statistically significant (OR ≈ 1.159, 95% CI [0.928–1.383], p = 0.206). These additional analyses are illustrated in a figure.


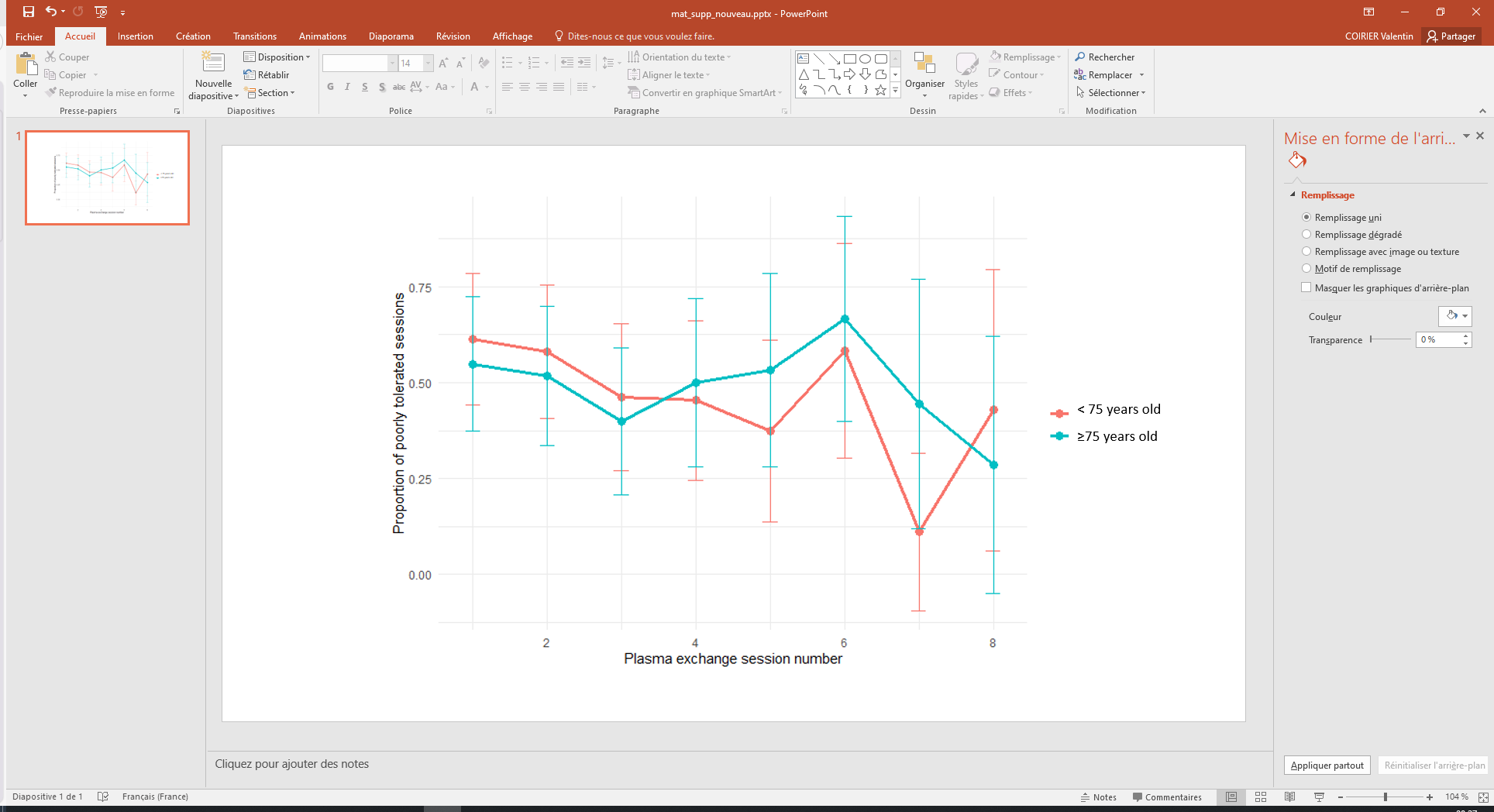

Supplement: Supplementary file 4 — Supporting Information 4: Description and results of the generalized estimating equations model. [file JCA-41-e70155-s004.docx]
